# Evaluation of the role of near-peer teaching in critical appraisal skills learning: a randomized crossover trial

**DOI:** 10.5116/ijme.5c39.b55b

**Published:** 2019-01-25

**Authors:** Indah S. Widyahening, Ardi Findyartini, Respati W. Ranakusuma, Esthika Dewiasty, Kuntjoro Harimurti

**Affiliations:** 1 Department of Community Medicine, Faculty of Medicine Universitas Indonesia, Indonesia; 2 Department of Medical Education, Faculty of Medicine Universitas Indonesia, Indonesia; 3 Clinical Epidemiology and Evidence Based Medicine (CE-EBM) Unit, Cipto Mangunkusumo Hospital,Faculty of Medicine Universitas Indonesia, Indonesia; 4 Department of Internal Medicine, Cipto Mangunkusumo Hospital, Faculty of Medicine Universitas Indonesia, Indonesia

**Keywords:** Peer teaching, evidence-based medicine, critical appraisal, undergraduate

## Abstract

**Objectives:**

The study sought to evaluate near-peer tutors’ teaching of critical appraisal skills to medical students as an aspect of Evidence-based Medicine.

**Methods:**

In a randomized crossover trial, 241 students completing a Clinical Epidemiology and Evidence-based Medicine (CE-EBM) module in the Faculty of Medicine Universitas Indonesia (FMUI) were randomly assigned to intervention or control groups. During tutorial sessions, intervention group participants were assigned to near-peer tutors, who were newly graduated doctors, and those in the control groups were assigned to staff tutors. After two tutorial sessions, intervention and control groups exchanged tutors for the next two sessions. Outcomes were measured using written knowledge and skills multiple choice questions (MCQ) test, the Evidence-based Practice Confidence Scale (EPIC) and a student attitude questionnaire, along with student evaluation of tutors to evaluate the process.

**Results:**

On completion of the module, the written test scores of intervention group students were similar to those of the control group (t_(239)_ = 1.553, p=0.122), as well as overall Evidence-based Practice Confidence Scale scores (F_(2/170)_ = 0.179, p = 0.673) and attitude scores (t_(219)_ =-0.676, p = 0.085). In the tutor evaluations, the students rated their near-peer tutored sessions as better than those tutored by staff in most respects.

**Conclusions:**

Near-peer tutors were as effective as and more readily accepted than staff tutors in teaching critical appraisal skills. These findings support the broader implementation of peer-teaching in other areas of medical education.

## Introduction

To process research findings and other information, medical doctors need critical appraisal skills for careful and systematic assessment of the trustworthiness, value, and relevance of the available evidence in particular contexts. Medical students must, therefore, be prepared to learn and master these skills, and various approaches have been implemented to that end.^1^^-^^3^ Within medical education, peer teaching, peer-assisted learning, peer tutoring, peer assessment, and other methods involving the deployment of students or trainees in a teaching role have attracted increasing interest.^4^^,^^5^ Peer teaching can be defined as “an educational arrangement in which one student teaches one or more fellow students.” When the teacher is a student who is more advanced (by at least one year) in the same curriculum as the learner, this is known as “near-peer teaching,” which is one of the most common forms of peer teaching.^4^ Some studies have also classified recently graduated medical doctors as near-peer teachers by virtue of their proximity in age and experience.^6^^,^^7^ Peer teaching and near-peer teaching methods have many advantages, which include alleviating faculty teaching burden, providing role models for junior students, enhancing students’ intrinsic motivation, and preparing physicians for their future role as educators.^5^ Although the effectiveness and efficiency of peer-assisted learning have been demonstrated in previous studies, these related mainly to a facilitation of psychomotor skills. Several of the published randomized controlled trials assessing the effectiveness of peer teaching relate to clinical skills such as intravenous (IV) access and bladder catheterization,^8^ neurological examination and lumbar puncture,^9^ resuscitation skills,^10^ interpretation of musculoskeletal ultrasound of the shoulder (as part of a sports medicine module),^11^ and basic clinical examination skills.^12^

Among the limited number of studies comparing student-led tutorials (SLT) and faculty-led tutorials (FLT), Kassab and colleagues^13^ studied the use of problem-based learning (PBL) to teach haematology. Other non-randomized controlled studies of peer teaching of evidence-based medicine include Josephson et al. in the US^14^ and Rees and colleagues in the UK.^15^ However, both of those aimed solely to demonstrate the feasibility of such programs rather than to provide evidence of the effectiveness of peer teaching interventions. Sabouni and colleagues ^16^ also explored the utilization of peer teaching in an evidence-based medicine course in a resource-poor setting. Although the knowledge and skills of course participants were found to have improved significantly after the course, the study did not report any change in attitude. In addition, the pre-post study design is not the most robust means of determining the effectiveness of a given intervention.

Although the literature notes many benefits of peer-assisted learning, these are largely confined to the acquisition of psychomotor skills. In light of the growing need for innovative ways of incorporating EBM into medical school curricula, especially where human resources are limited, the present study aimed to evaluate the effectiveness of recently graduated medical doctors as near-peer tutors as compared to experienced medical staff. Specifically, we hypothesized that near-peer tutors would be as effective as medical staff in teaching critical appraisal skills to medical students as part of a Clinical Epidemiology and Evidence-based Medicine (CE-EBM) program.

## Methods

### Study design and participants

The study was designed as a randomized crossover trial. The participants were fourth-year medical students completing the CE-EBM module in the Faculty of Medicine Universitas Indonesia (FMUI) in the academic year 2014–2015. As the CE-EBM module is compulsory for fourth-year students, the entire cohort of 241 students participated. The module was conducted in two rotations: 116 students in rotation 1 (May–June 2015) and 125 students in rotation 2 (June–July 2015).  The crossover design meant that every student had an equal opportunity of being tutored by staff members or recently graduated medical doctors. The Research Ethics Committee of FMUI granted ethical clearance.

### CE-EBM module and intervention

The content and structure of the CE-EBM module at FMUI has been described elsewhere.^17^^,^^18^ Adapted from the University Medical Center Utrecht, the module is completed over four weeks. Informed by the principles of the Sicily statement on EBM teaching,^19^ the module includes lectures, tutored group discussions, and moderated plenary presentations on the design and conduct of diagnostic, therapeutic, prognostic, and etiologic studies, as well as computer-based literature search and data analysis.

Skills for critical appraisal of scientific papers were practiced during four 2-hour tutored group discussions. Journal articles on diagnosis, therapy, prognosis, or etiologic studies were provided by the module coordinators to ensure that the studies in question were well designed and could be appraised by students at this level. Led by a tutor, the participating students undertook a critical appraisal of the studies in their respective groups, using the worksheets provided by the Oxford Centre for EBM.^20^ Prior to these discussions, the students attended corresponding lectures delivered by experts in clinical epidemiology and evidence-based medicine.

The voluntary near-peer tutors were recruited from FMUI’s newly graduated medical doctors, who had passed the CE-EBM module in their fourth year and had been practising EBM during their clerkship. The selected candidates were trained in a three-day training of teachers (TOT) that focused on tutoring skills and group dynamics. These new graduates were selected as near-peer tutors on the basis of their level and proximity in age to the fourth-year medical students. Five recently graduated medical doctors who were awaiting internship placement ultimately participated as near-peer tutors (of whom four participated in both rotations while one participated only in the first rotation).

The control groups were taught by experienced medical staff who had already participated in a two-day EBM course conducted by FMUI or in a three-day EBM course by Oxford CEBM and were practicing EBM in their clinical work. In total, 15 medical staff from various departments participated as tutors (seven in rotation 1 and eight in rotation 2), having attended a similar three-day TOT on EBM.

**Figure 1:**
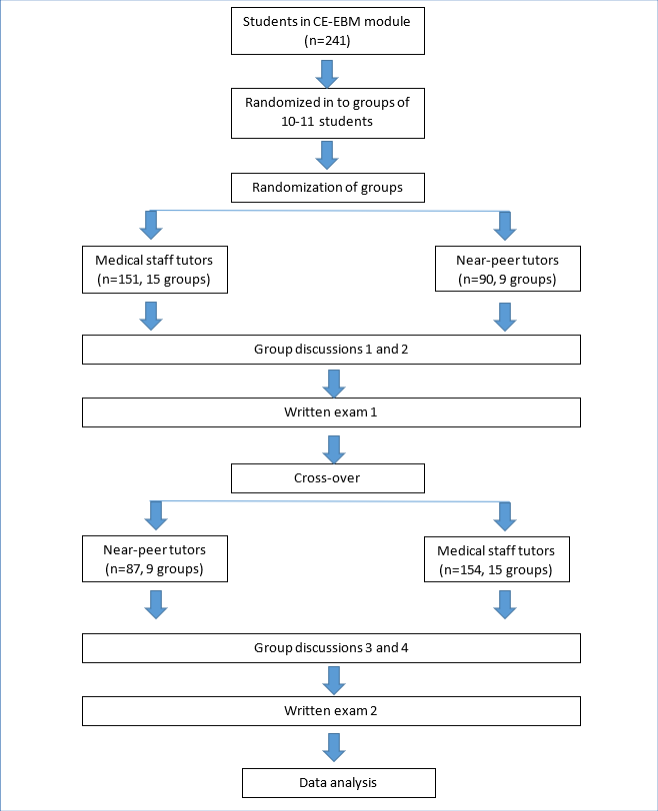
The flow-chart of study

The students were randomly assigned to groups of 10 or 11. For the first two group discussion sessions, each group was randomly scheduled to be tutored either by medical staff or junior doctors. During the last two group discussion sessions, the groups were crossed over (see Figure 1). Randomization was implemented using computer software. As medical staff tutors and near-peer tutors were unequal in number, five groups did not participate in the crossover and were tutored by medical staff throughout.

### Data collection

**Figure 2:**
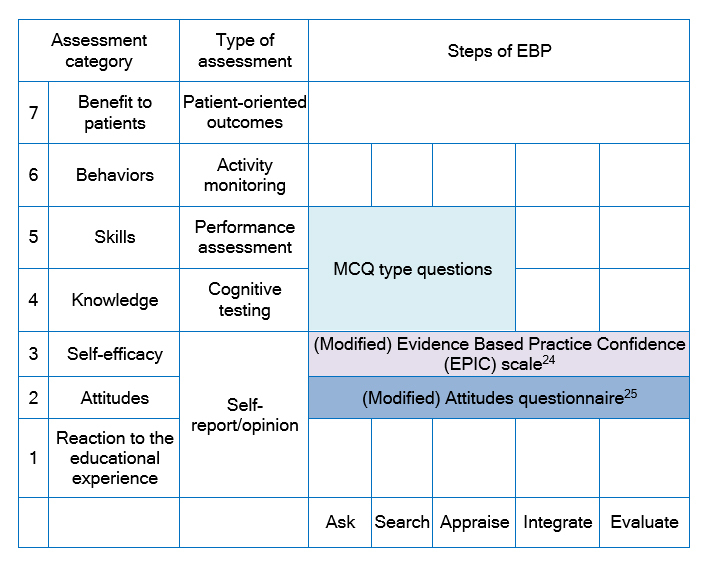
The Classification Rubric for EBP Assessment Tools in Education (CREATE) framework21 for the Faculty of Medicine Universitas Indonesia (FMUI) Clinical Epidemiology and Evidence-Based Medicine module evaluation

Evaluation of the teaching strategies was conducted in accordance with the Classification Rubric for Evidence-based Practice Assessment Tools in Education (CREATE) framework^21^ as shown in Figure 2.

Student knowledge and skills were evaluated using a written test comprising 30 multiple choice questions (MCQ) with five options (similar to the Berlin questionnaire^22^ and Fresno test^23^ as validated assessments of EBM skills). The CE-EBM faculty team developed the questions, based on predefined learning outcomes and in line with the national standard for medical education assessment in Indonesia to ensure the content and face validity of the test as an educational measurement tool. The test was conducted twice; the first (mid-test, after the first two discussion sessions) pertained to clinical question formulation, search, diagnosis, and prognosis, and the second (final test, after the last two sessions) related to therapy, etiology, and systematic review.

Evaluation of self-efficacy utilized a modified version of the Evidence-based Practice Confidence (EPIC) scale;^24^ students rated their level of confidence in their ability to perform each evidence-based practice (EBP) activity on an 11-point scale ranging from 0% (cannot do at all) to 100% (certain can do). Item-level responses were averaged to obtain a summary score, which ranged from 0% to 100%. The evaluation was completed at the beginning and end of the module. Cronbach’s α was computed to assess the internal consistency of the EPIC questionnaire during baseline (pre-module) assessment. The pre-module Cronbach’s α value for the EPIC questionnaire was 0.95.

Attitudes were measured using part of the Knowledge Attitudes and Behaviors questionnaire developed by Johnston and colleagues,^25^ which had previously been used to assess the first implementation of the CE-EBM module in FMUI and had been validated accordingly, with a Cronbach’s α value of 0.76.^18^ Students were asked to score each item on a 6-point Likert scale (from strongly agree to strongly disagree). The attitude questionnaire was also administered at the beginning and end of the module.

Finally, student educational experience was also evaluated, although EBP steps were not explicitly measured. This final questionnaire assessed more general aspects of the tutorial process as evaluated by students; it was previously utilized by Knobe and colleagues^11^ to evaluate the effectiveness of peer-assisted teaching of technical skills for interpreting ultrasound images of the shoulder. Students rated their experience using a 5-point Likert scale, ranging from 1 (strongly disagree) to 5 (strongly agree). All evaluations were conducted after the first two discussion sessions and again after the last two sessions following crossover.

### Data analysis

The primary outcome measure was the difference in mean score between intervention and control groups; independent t-testing was used to assess the significance of any discrepancies. For outcomes that differed significantly from baseline scores, analysis of covariance (ANCOVA) was utilized, using the baseline score as a covariate. The analysis was based on the intention-to-treat principle. All analyses were performed using SPSS version 22.0.

## Results

The mean + SD age of participating students was 22.1+1.1 years (range 18.9–26.7 years), of whom 89 (36.9%) were male. The median age of near-peer tutors was 24 years (range 23–26 years). The median age of staff tutors was 41.5 years (range 37–55 years).

**Table 1:** Attitudes of the Faculty of Medicine Universitas Indonesia (FMUI) 4th-year medical students on Evidence-Based Practice (n=241)

Attitudes item	Before the module				After the module			
	Near-peer-tutored (n=127)	Staff-tutored (n=68)	t	p	Near-peer-tutored (n=161)	Staff-tutored^*^ (n=59)	t	p
1. Evidence-based medicine is “cook-book” medicine that disregards clinical experience.	3.17 (0.91)	3.15 (0.96)	-0.098	0.922	2.98 (1.20)	2.70 (1.05)	-1.575	0.117
2. There is no reason for me personally to adopt evidence-based medicine because it is just a “fad” (or “fashion”) that will pass with time.	2.61 (0.88)	2.5 (1.0)	-0.764	0.446	2.49 (1.03)	2.43 (0.90)	-0.452	0.651
3. If evidence-based medicine is valid, then anyone can see patients and do what doctors do.	3.32 (1.19)	3.07 (1.21)	-1.386	0.167	3.42 (1.42)	2.85 (1.29)	-2.732	0.007
4. Evidence-based medicine ignores the “art” of medicine.	2.82 (0.89)	2.75 (0.87)	-0.521	0.603	2.61 (1.07)	2.54 (0.89)	-0.449	0.654
5. Doctors, in general, should not practice evidence-based medicine because medicine is about people and patients, not statistics.	2.8 (0.77)	2.62 (0.75)	-1.620	0.107	2.53 (1.06)	2.43 (0.92)	-0.681	0.497
6. Previous work experience is more important than research findings in choosing the best treatment available for a patient.	3.10 (0.75)	3.09 (0.88)	-0.118	0.906	2.94 (1.01)	2.73 (0.94)	-0.661	0.169
7. Overall score	2.97 (0.53)	2.85 (0.61)	-1.435	0.153	2.83 (0.79)	2.62 (0.73)	-0.676	0.085

^*^Consisted of groups of students who were not assigned to the cross-over as well, thus the respective groups were subject to near-peer tutor during the whole duration of the module.

**Table 2:** Comparison of the Evidence-Based Practice Confidence (EPIC) Scale of the Faculty of Medicine Universitas Indonesia (FMUI) 4th medical students (n=241)

Evidence-based Practice activity	Before the module				After the module			
	Near-peer-tutored (n=108)	Staff-tutored (n=66)	t	p	Near-peer-tutored (n=161)	Staff-tutored† (n=65)	t/F	p
How confident are you in your ability to:								
1. Identify a gap in your knowledge related to a patient or client situation (e.g. history, assessment, treatment)?	61.20 (13.09)	61.21 (15.04)	0.004	0.997	72.17 (11.55)	72.46 (13.92)	0.159	0.873
2. Formulate a question to guide a literature search based on a gap in your knowledge?	63.52 (15.12)	60.00 (16.82)	-1.427	0.156	78.26 (10.64)	79.23 (9.891)	0.633	0.528
3. Effectively conduct an online literature search based on a gap in your knowledge?	65.83 (14.08)	63.64 (16.04)	-0.947	0.345	77.95 (10.43)	78.77 (10.38)	0.535	0.593
4. Critically appraise the strengths and weaknesses of study methods (e.g. appropriateness of study design, recruitment, data collection and analysis)?	54.44 (16.87)	46.97 (20.97)	-2.451	0.016	75.28 (9.88)	72.19 (10.15)	0.003	0.956*
5. Critically appraise the measurement properties (e.g. reliability and validity, sensitivity and specificity) of standardized tests or assessment tools you are considering using in your practice?	53.36 (15.83)	48.64 (20.22)	-1.618	0.108	76.38 (9.74)	73.69 (11.26)	-1.788	0.075
6. Interpret study results obtained using statistical tests such as t-tests or chi-square test)?	56.98 (18.46)	53.03 (20.75)	-1.304	0.194	72.38 (14.12)	71.69 (12.69)	-0.338	0.736
7. Interpret study results obtained using statistical procedures such as linear or logistic regression?	48.89 (18.81)	45.30 (20.32)	-1.183	0.238	66.96 (15.17)	63.54 (15.95)	-1.511	0.132
8. Determine if evidence from the research literature applies to your patient's or client's situation?	60.75 (15.16)	52.88 (21.32)	-2.617	0.010	75.13 (10.64)	74.84 (10.23)	0.508	0.477*
9. Ask your patient or client about his/her needs, values and treatment preferences?	67.50 (13.47)	63.03 (19.13)	-1,663	0.099	77.13 (11.62)	77.54 (10.76)	0.247	0.805
10. Decide on appropriate course of action based on integrating the research evidence, clinical judgement and patient or client preferences?	60.74 (15.32)	55.61 (18.32)	-1.905	0.059	75.60 (11.11)	74.77 (10.62)	-0.513	0.609
11. Continually evaluate the effect of your course of action on your patient's or client's outcomes?	61.85 (13.95)	58.18 (19.12)	-1.354	0.179	75.96 (11.26)	75.54 (11.32)	-0.256	0. 798
Overall	59.56 (11.62)	55.32 (15.77)	-1.895	0.061	74.82 (8.49)	74.04 (8.65)	0.179	0.673*

^*^ANCOVA with baseline score as covariate †Consisted of groups of students that were not being assigned to the cross-over as well. Thus, the respective groups were not being subject to near-peer tutor during the whole duration of the module.

**Table 3:** Assessment of the Faculty of Medicine Universitas Indonesia (FMUI) Clinical Epidemiology and Evidence-based Medicine (CE-EBM) module tutors by the 4th year medical students (n=241)

Item of assessment	Mean (SD)		t	p
	Near-peer tutored	Staff-tutored		
Tutorial 1 and 2	n=87	n=143		
1. The tutor was competent	4.13 (0.71)	4.10 (0.71)	-0.223	0.824
2. The lessons were enjoyable	4.36 (0.68)	4.12 (0.85)	-2.206	0.028
3. I was able to learn a lot	4.18 (0.69)	3.87 (0.88)	-2.802	0.006
4. I was able to directly apply what I learned	4.00 (0.73)	3.76 (0.92)	-2.227	0.027
5. Theory and practice were well combined	4.06 (0.81)	3.83 (0.80)	-2.052	0.041
6. I would rather have been in a different group	1.92 (1.21)	1.85 (1.18)	-0.452	0.652
7. Group size was optimal	4.20 (0.76)	4.15 (0.88)	-0.427	0.670
8. Interaction between students and teacher was good	4.49 (0.66)	4.27 (0.74)	-2.283	0.023
9. There were many unanswered questions	2.43 (1.01)	2.86 (1.10)	2.992	0.003
10. Time was tight	2.90 (1.14)	2.84 (1.19)	-0.360	0.719
11. The topic was too complex	2.87 (1.09)	2.75 (1.02)	-0.879	0.380
Overall	3.59 (0.41)	3.49 (0.36)	-2.009	0.046
Tutorial 3 and 4	n=83	n=150		
1. The tutor was competent	4.40 (0.68)	4.09 (0.79)	-2.958	0.003
2. The lessons were enjoyable	4.51 (0.61)	4.07 (0.95)	-4.218	<0.001
3. I was able to learn a lot	4.42 (0.66)	3.76 (0.97)	-6.130	<0.001
4. I was able to directly apply what I learned	4.14 (0.72)	3.61 (0.88)	-4.980	<0.001
5. Theory and practice were well combined	4.22 (0.70)	3.67 (0.92)	-5.052	<0.001
6. I would rather have been in a different group	2.08 (1.40)	2.09 (1.26)	0.013	0.99
7. Group size was optimal	4.24 (0.81)	4.26 (0.72)	0.156	0.876
8. Interaction between students and teacher was good	4.51 (0.63)	4.20 (0.83)	-2.973	0.003
9. There were many unanswered questions	2.38 (1.24)	2.80 (1.10)	2.658	0.008
10. Time was tight	2.52 (1.32)	2.58 (1.17)	0.332	0.741
11. The topic was too complex	2.66 (1.19)	2.80 (0.98)	0.975	0.330
Overall	3.65 (0.50)	3.45 (0.41)	-3.237	0.001

A mid-evaluation following the first two discussion sessions found no statistically significant difference (t_(__241)_ = -1.520, p = 0.130) between mean + SD of written test scores for the near-peer tutored group (65.89 + 12.86) and the staff-tutored group (63.18 + 13.69). The groups then exchanged tutors for the next two discussion sessions. At the end of the module, the difference in mean + SD of written test scores for the near-peer tutored group (68.20 + 12.50) and the staff-tutored group (70.61+10.99) was again statistically non-significant (t_(__241) _= 1.553, p = 0.122).

At the beginning of the module, student attitude scores were comparable (Table 1). After the module, there were no statistically significant differences in the mean overall attitude score or for the majority of attitude items other than the statement “If evidence-based medicine is valid, then anyone can see patients and do what doctors do.”

The overall score for level of student confidence in Evidence-based Practice (based on the EPIC questionnaire) at the beginning of the module was also comparable for both groups (Table 2). However, a statistically significant lower score was observed for medical staff on two items (4 - Critically appraise the strengths and weaknesses of study methods, and 8 - Determine if evidence from the research literature applies to your patient's or client's situation). No statistically significant difference in mean EPIC scale score was observed either for individual items or overall scores at the end of the module. When the students were asked to evaluate the tutorial process, they assigned more positive scores to the near-peer tutor for the majority of evaluation items (Table 3).

## Discussion

In the present study, near-peer tutored students and staff-tutored students achieved comparable scores on knowledge, skills, attitudes, and confidence in relation to critical appraisal skills. Although confirming what has been reported in earlier studies, these findings must nevertheless be interpreted with a degree of caution. For example, the similarity of these results, especially on the written test, may reflect the fact that all students attended lectures delivered by experts in clinical epidemiology. Another possible explanation (as suggested by ten Cate and colleagues)^26^ is that peer-teaching may also encourage students to invest more effort in the study because of concerns about inadequate teaching.

Additionally, students scored better on the tutorials led by near-peer tutors than on those led by medical staff tutors. Tolsgaard and colleagues reported a similar result in a study employing second-year medical students as tutors to teach IV access and bladder catheterization to first-year medical students at the University of Copenhagen.^8^ As explained by cognitive congruence theory, a teacher whose knowledge base is similar (i.e., congruent) to the learner’s is likely to be more effective than an expert in the field who has a dissimilar knowledge base (i.e., who is cognitively incongruent or is perceived as more “cognitively distant”). Peer-tutors are also believed to be able to provide a more comfortable and safe learning environment for students, as they come from a similar social environment.^5^

A systematic review by Burgess and colleagues^27^ indicated that practical issues such as limited faculty resources were most frequently reported to account for the implementation of peer or near-peer teaching. Another justification for the use of near-peer tutors is the need to develop the teaching skills of future doctors. While the Indonesian Medical Council does not explicitly list teaching skills among the competencies of medical doctors, Indonesian medical graduates are expected to be able to provide health education to patients, families, and communities.^28^ In Tomorrow’s Doctors, the UK General Medical Council states that medical graduates must be able to demonstrate appropriate teaching skills,^29^ and the Royal College of Physicians and Surgeons of Canada states in its CanMEDS Physicians Competency framework that the physician’s role includes being a “scholar” who are competent to “teach students, residents, the public, and other health care professionals”.^30^

As well as the benefits for the medical students, the peer tutors also gained, as the teaching experience motivated them to learn more and to deepen their understanding of the subject. This is an expected aspect of EBM skills, which are currently seen as one of the core competencies of medical doctors. Experience in teaching and preparation of teaching materials is also known to build leadership capability and confidence.^5^

Although peer teaching has been widely implemented in medical education, we are not aware of any previous studies of this kind from Indonesia, although in our experience the practice is undoubtedly performed informally. We recognize that as educational interventions are generally complex, it is often difficult to distinguish which element accounts for which effect or to what extent it might be adapted to local circumstances while remaining effective.^31^ Moreover, certain particulars may limit the generalization of the present findings, such as student characteristics and EBM as a distinctive subject. As the oldest and most prominent medical school in Indonesia, FMUI students are a select group, and earlier research found that tutor expertise did not affect FMUI students’ performance during problem-based learning.^32^ Furthermore, the ethics of medical education require the minimization of any potential disadvantage to students assigned to a near-peer tutor – hence the need for a crossover. As a result, none of the students in this study were assigned only to a near-peer tutor, and this may have led to over-estimation of results, especially in the final test. Future studies should, therefore, extend the utilization of near-peer tutors to all tutorial sessions.

## Conclusions

The present findings confirm that near-peer tutors can be as effective as and more readily accepted than staff tutors. This is based on the performance of students in critical appraisal skills in EBM, extending the evidence of the benefits of peer-assisted learning beyond the area of psychomotor skills. This study also provides preliminary evidence that medical school educational policy will be accommodated to the assignment of trained near-peer tutors to teach critical appraisal skills to medical students.

### Acknowledgements

We would like to express our gratitude to the Dean, the Vice Dean for Education, Research, and Student Affairs and the Manager of Education and Student Affairs at FMUI for lending their full support to this study. The study received full financial support from the Medical Education Unit of FMUI.

### Conflict of Interest

The authors declare that they have no conflict of interest.
